# Tumour infiltrating lymphocytes and immune-related genes as predictors of outcome in pancreatic adenocarcinoma

**DOI:** 10.1371/journal.pone.0219566

**Published:** 2019-08-05

**Authors:** Alberto D’Angelo, Navid Sobhani, Giandomenico Roviello, Stefan Bagby, Deborah Bonazza, Cristina Bottin, Fabiola Giudici, Fabrizio Zanconati, Nicolo De Manzini, Alessandra Guglielmi, Daniele Generali

**Affiliations:** 1 Department of Biology and Biochemistry, University of Bath, Bath, United Kingdom; 2 Department of Medical, Surgical, & Health Sciences, University of Trieste, Piazza Ospitale, Trieste, Italy; 3 Breast Cancer Unit, ASST Cremona, Cremona, Italy; 4 Department of Medical Sciences, Ospedale di Cattinara, Università degli Studi di Trieste, Strada di Fiume, Trieste, Italy; Vrije Universiteit Brussel, BELGIUM

## Abstract

**Background:**

We investigated the correlation between pancreatic ductal adenocarcinoma patient prognosis and the presence of tumour infiltrating lymphocytes and expression of 521 immune system genes.

**Methods:**

Intratumoural CD3+, CD8+, and CD20+ lymphocytes were examined by immunohistochemistry in 12 PDAC patients with different outcomes who underwent pancreaticoduodenectomy. The results were correlated with gene expression profile using the digital multiplexed NanoString nCounter analysis system (NanoString Technologies, Seattle, WA, USA).

**Results:**

Twenty immune system genes were significantly differentially expressed in patients with a good prognosis relative to patients with a worse prognosis: *TLR2* and *TLR7* (Toll-like receptor superfamily); *CD4*, *CD37*, *FOXP3*, *PTPRC* (B cell and T cell signalling); *IRF5*, *IRF8*, *STAT1*, *TFE3* (transcription factors); *ANP32B*, *CCND3* (cell cycle); *BTK* (B cell development); *TNF*, *TNFRF1A* (TNF superfamily); *HCK* (leukocyte function); *C1QA* (complement system); *BAX*, *PNMA1* (apoptosis); *IKBKE* (NFκB pathway). Differential expression was more than twice log 2 for *TLR7*, *TNF*, *C1QA*, *FOXP3*, and *CD37*.

**Discussion:**

Tumour infiltrating lymphocytes were present at higher levels in samples from patients with better prognosis. Our findings indicate that tumour infiltrating lymphocyte levels and expression level of the immune system genes listed above influence pancreatic ductal adenocarcinoma prognosis. This information could be used to improve selection of best responders to immune inhibitors.

## Introduction

Pancreatic ductal adenocarcinoma (PDAC) is a lethal disease often with a poor prognosis [1]. PDAC is characterized by a desmoplastic, highly heterogeneous and immune-suppressive microenvironment that hinders antitumour immunity. PDACs tend to recruit immunosuppressive cells including myeloid-derived suppressor cells (MDSCs), regulatory T cells (Tregs) and tumour-associated macrophages (TAMs). PDACs also inhibit immune effector cells, mainly CD4^+^ CD8^+^ T lymphocytes, natural killer (NK) cells and dendritic cells (DCs) [2]. Some PDACs, moreover, overexpress programmed death ligand 1 (PDL1) and secrete inhibitory cytokines such as interleukin 10 (IL10) and tumour growth factor β (TGFβ) [3]. Desmoplasia, another hallmark of PDAC, is characterized by a noticeable proliferation of myofibroblasts and generates a strong barrier against tumour infiltration by both immune cells and drugs [4].

The correlation between tumour infiltrating lymphocytes (TILs) and clinical outcomes has been investigated in several studies involving PDAC patients. Fukunaga et al found that patients with CD8^+^ tumours had a higher five-year overall survival (OS) rate than patients with CD8^-^ tumours, and similarly for patients with CD4^+^ tumours versus CD4^-^ tumours [5]. Evaluating the prognostic value of CD3^+^, CD8^+^, and CD20^+^ TILs, Tewary et al found an association between CD3^+^ and CD20^+^ TILs and a higher survival rate [6]. Furthermore, Ino et al found that patients with high CD8^+^ and CD4^+^ TIL and low Treg counts had a better survival rate than patients with low CD8^+^ and CD4^+^ TIL and high Treg counts [7]. Patients with CD4^+^ tumours were found to have OS and disease-free survival (DFS) higher than patients with CD4^-^ tumours, and a high CD8^+^/FoxP3^+^ lymphocyte ratio correlated with better clinical outcomes, but no significant correlation was found between CD8^+^ TILs and survival or other clinical-pathological features [8]. Karakhanova et al found that CD4^+^ and CD8^+^ count correlated with higher OS and DFS [9].

The immune system interacts intimately with tumours over the entire process of disease development. This complex immune system-tumour cross-talk can both inhibit and enhance tumour growth [10]. Major features of the process by which tumours escape the immune system include a reduction in the recognition of cancer antigens by immune cells and the development of an immune suppressive microenvironment. The tumour microenvironment of PDAC has been consistently reported to be capable of promoting immune escape, rendering the immune system ineffective in eliciting an anti-tumour response. Improved understanding of the tumour microenvironment and how it contributes to immune evasion could, therefore, lead to better treatments and outcomes for PDAC patients [11]. This motivated our investigation of differences in TILs and immune-related gene expression between PDAC patients with good and worse clinical outcomes.

## Materials & methods

### Patients and sample collection

Fresh PDAC specimens were obtained from patients (n = 12) undergoing surgical resection at the Department of Medical, Surgical & Health Sciences, Cattinara Teaching Hospital, Trieste University, between 2005 and 2015. These experiments were approved by Trieste University Institutional Review Board. Tissue specimens were snap frozen in liquid nitrogen and stored at −80°C.

Formalin-fixed, paraffin wax-embedded sections were used for immunohistochemical staining. All 12 paraffin wax blocks were confirmed to contain tumour tissue by two pathologists, comprising six pancreatic adenocarcinomas with a good prognosis and six pancreatic adenocarcinomas with a bad prognosis.

The following clinical data were collected: patient age, gender, and outcome; the presence/absence of metastasis; tumour location, size, margin status, TNM stage, degree of differentiation, invasion degree and location (lymph node, bile duct/duodenal serosa, hepatic, portal vein, vascular, perineural), schedule of chemotherapy, neoadjuvant and/or adjuvant chemotherapy, chemotherapy toxicity, and treatment follow up. Clinical Stadiation of patients ranged from stage IA to III but none of them was metastatic and none of them underwent chemotherapy. Patients were informed about the project and gave written consent for study participation. Of note, a caveat to the study is that sample was relatively scarce because of the intrinsically hard-to-reach nature of the disease plus the requirement to put aside part of every sample for storage at the institution as per legal and ethical regulations.

### Follow up

**Fig 1:**
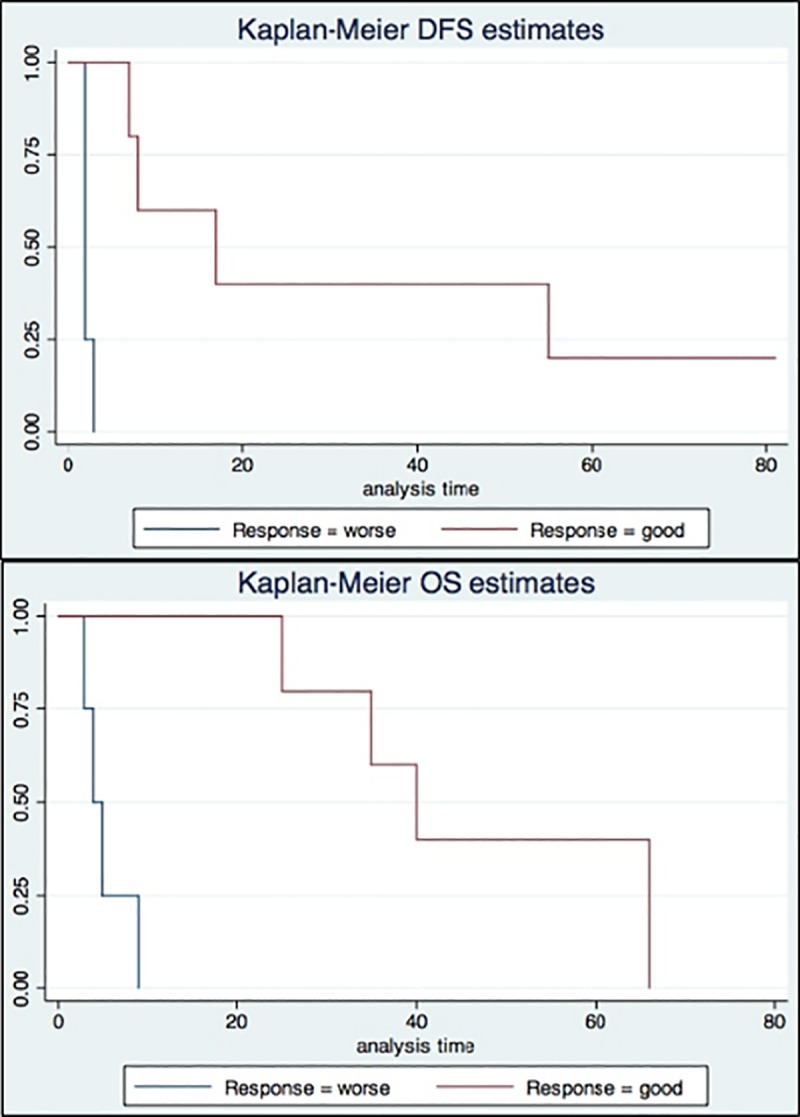
Worse and good prognosis PDAC patient groups. Kaplan-Meier curves show the difference of OS or DFS between two groups of patients: one group with a worse prognosis (“worse”, blue line) and the other with a better prognosis (“good”, red line).

OS was measured from the time of surgery to the time of death or the last follow up visit. Dates of death were obtained from patient hospital records or follow up telephone calls. A more in-depth analysis of the 12 patients revealed two groups with different DFS and/or OS: six patients with an OS between 25 and 66 months were classified as “good cases”, while six with OS between 2 and 9 months or DFS between 1 and 2 months were classified as “worse cases”. Table 1 summarizes the clinical-pathological data of the two groups of patients and Fig 1 shows the Kaplan-Meier curves for DFS and OS for the two groups of patients.

**Table 1:** Summary of patient clinical-pathological information. ECOG: Eastern Cooperative Oncology Group (Performance Status); TNM Stadiation: T = Tumour (0–4), N = Nodes (0–2), M = Metastasized (0–1).

	Good cases	Worse cases
**Num. Pts**	6	6
**Age**	65.3	63.8
**Gender**		
Male	4	3
Female	2	3
**ECOG**		
0	2	0
1	2	3
2	1	2
Not reported	1	1
**TNM**		
**T**		
T0	0	0
T1	1	2
T2	0	4
T3	4	0
T4	1	0
**N**		
N0	2	2
N1	4	4
**M**		
M0	6	6
STAGE	IA/IIA/IIB/III	IB/IIA/IIB

### PanCancer immune profile panel multiplex gene expression profiling

Three patients were randomly selected from each of the good and worse prognosis groups. In accord with *Koti et al* [12], pathologists in our department selected and extracted two areas of at least 70% of cellularity from each formalin-fixed, paraffin wax-embedded tumour tissues, and total RNA was extracted using Qiagen RNeasy (Qiagen Inc., Toronto, ON, Canada) as per the manufacturer’s instructions. A NanoDrop ND-100 spectrophotometer (NanoDrop Technologies, Wilmington, DE, USA) was used to measure RNA concentration and purity. All RNA samples included in the study passed the quality control requirements (as verified by RNA integrity number or OD 260/280 ratio) of the platform. Using 100 ng total RNA from each sample as input, according to the manufacturer’s instructions, the digital multiplexed NanoString nCounter analysis system (NanoString Technologies, Seattle, WA, USA) was used for gene expression profiling. Tumour RNA samples were analysed using nCounter PanCancer immune profile panel consisting of 770 human immune-related genes (Nanostring Technologies).

In this assay, colour-coded barcodes are used to represent single-target transcripts in the reaction. An overnight hybridisation reaction was used to incorporate the resulting material, carried out by combining 20 ml of nCounter Reporter probes in hybridisation buffer, 5 ml of nCounter Capture probes and 5 ml of the total RNA sample for a total reaction volume of 30 ml. The hybridisations were incubated at 65°C for 16–20 h. An excess of probes is provided during overnight hybridization to ensure that each target finds a probe pair. Target abundance values can then be determined through the nCounter Digital Analyzer by counting the individual fluorescent barcodes. A high-density scan was performed for each assay (encompassing 600 fields of view). After hybridisation, the cartridges were analysed in the Digital Analyzer that counts (representing the number of molecules) and arranges the barcodes.

### Immunohistochemistry

An automatic stainer (BenchMark ULTRA, Ventana Medical System, Inc.) was used for the immunohistochemical test. The antigen was retrieved with cell conditioning buffer 1. Next, endogenous peroxidase was inhibited with H_2_O_2_ at 3% (Bioptica) for 10 min. Samples were incubated with primary antibody anti-CD3 (2GV6) (Roche-Ventana), anti-CD8 (SP57) (Roche-Ventana), Rabbit Monoclonal Pre-diluted (0.4μg/mL), for 20 min at 36°C; anti-CD20 (L26) (Roche-Ventana), Mouse Monoclonal Pre-Diluted (0.4μg/mL) for 24 min at 36°C. The antibody was exposed with ultraView Universal DAB Detection Kit (Cat No. 760–500). As counterstain, Mayer haematoxylin was used for 4 min.

TIL levels were assessed by two investigators blind to the patients’ clinical-pathological data using the standardized method coded in 2015 by the International TILs Working Group [13]. TILs were investigated per microscopic field (5X and 10X) and an average over ten independent regions having the most abundant immunoreactive cells was calculated for each slide.

### Immunohistochemistry statistical analysis

For immunohistochemistry statistical analysis, a preliminary data exploration was performed. Numerical variables were expressed as median and range and were compared by non-parametric tests (Mann–Whitney U-test). Qualitative data were expressed as frequencies and organized into contingency tables; the association between categorical variables was investigated by means of Fisher’s exact test or Pearson’s Chi-square. Time-dependent variables were calculated according to the Kaplan–Meier method. For the entire statistical analysis, the significance levels were established at p<0.05. All data were analysed with STATA software.

### Immune profile panel multiplex nanoString statistical analyses

#### NanoString data analysis

nSolver (NanoString Technologies) was used for the normalization of raw data as previously reported [14]. The raw NanoString counts were initially subjected to normalization for all target RNAs in all samples based on built-in positive controls. This step accounts for post-hybridization processing, inter-sample and experimental variation such as hybridization efficiency. The geometric mean of each of the controls was calculated, indicating the overall assay efficiency. For the mRNA content normalisation, housekeeping genes were then used. To facilitate downstream statistical analysis, values < 0 were blanketed and considered equal to 1. After initial normalisation steps, data were imported to GraphPad Prism (GraphPad Software, Inc., La Jolla, CA, USA) to be processed for statistical analysis. Multiple t-tests were performed with correction for multiple comparisons through the Sidak–Bonferroni method. A difference in expression with a P-value of ≤ 0.05 was considered statistically significant.

## Results

### Immune-related gene expression analysis in pancreatic adenocarcinomas with good versus worse prognosis

**Fig 2:**
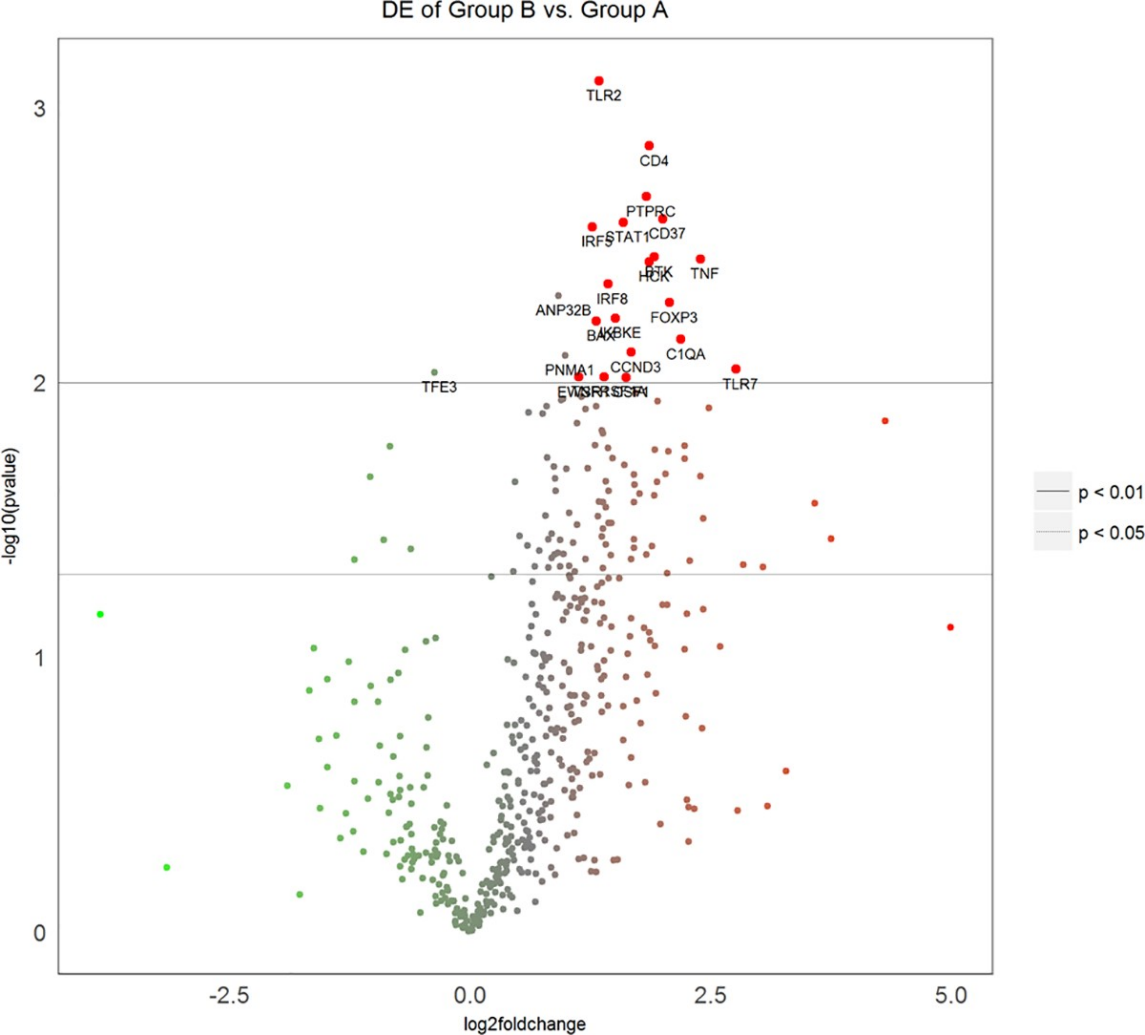
Differential gene expression between “good prognosis cases” and “worse prognosis cases” of PDAC. Volcano plot in which the larger red dots represent only those genes whose expression is at least twice log2 higher in “good cases” (Group B) relative to “worse cases” (Group A) with p-value < 0.01. Genes with highly statistically significant differential expression fall at the top of the plot above the horizontal lines, and highly differentially expressed genes fall to either side depending on whether they are negatively or positively differentially expressed. Horizontal lines indicate various False Discovery Rate (FDR) thresholds or p-value thresholds if there is no adjustment to the p-values. Genes are red if the resulting p-value is below the given FDR or p-value threshold. The 20 genes showing the most statistically significant differential expression are labelled in the plot.

Prognosis of primary PDAC patients was determined using clinical data and Kaplan-Meier curves (Fig 1). Three primary PDAC patients with a good prognosis and three with a worse prognosis were then chosen for mRNA analysis by PanCancer Immune Profile Panel multiplex gene expression analysis. Fig 2 shows the differential gene expression. Among the immune system genes showing statistically significant (p <0.01) differential expression between pancreatic adenocarcinoma with a good and worse prognosis, differential expression of *TLR7*, *TNF*, *C1QA*, *FOXP3*, and *CD37* was at least twice log 2: +2.76 log 2 ± 0.58 (p <0.00896), +2.39 log 2 ± 0.389 (p <0.00356), +2.19 log 2 ± 0.43 (p <0.00697), +2.07 log 2 ± 0.372 (p <0.00513), and +2 log 2 ± 0.297 (p <0.00254), respectively (S1 Fig). *BTK* (+1.91 log 2 ± 0.309 (p<0.0035)), *CD4* (+1.86 log 2 ± 0.235 (p<0.00138)), *HCK* (+1.86 log 2 ± 0.304 (p<0.00364)), *PTPRC* (+1.83 log 2 ± 0.259 (p<0.00211)), *CCND3* (+1.67 log 2 ± 0.337 (p<0.00777)), *STAT1* (+1.59 log 2 ± 0.238 (p<0.00626)), *IKBKE* (+1.51 log 2 ± 0.282 (p< 0.00585)), *IRF8* (+1.43 log 2 ± 0.246 (p<0.00439)), *TNFRF1A* (+1.39 log 2 ± 0.298 (p<0.00954)), *TLR2* (+1.34 log 2 ± 0.147 (p<0.000799)), *BAX* (+1.31 log 2 ± 0.246 (p<0.00598)), *IRF5* (+1.27 log 2 ± 0.193 (p<0.00272)), *PNMA1* (+0.986 log 2 ± 0.201 (p<0.00799)), *ANP32B* (+0.92 log 2 ± 0.163 (p<0.00484)), *TFE3* (-0.37 log 2 ± 0.0783 (p<0.00919)), and mRNA also showed statistically significant (p <0.01), but less than twice log 2, differential expression between pancreatic adenocarcinomas with good and worse prognosis (Table 2).

**Fig 3:**
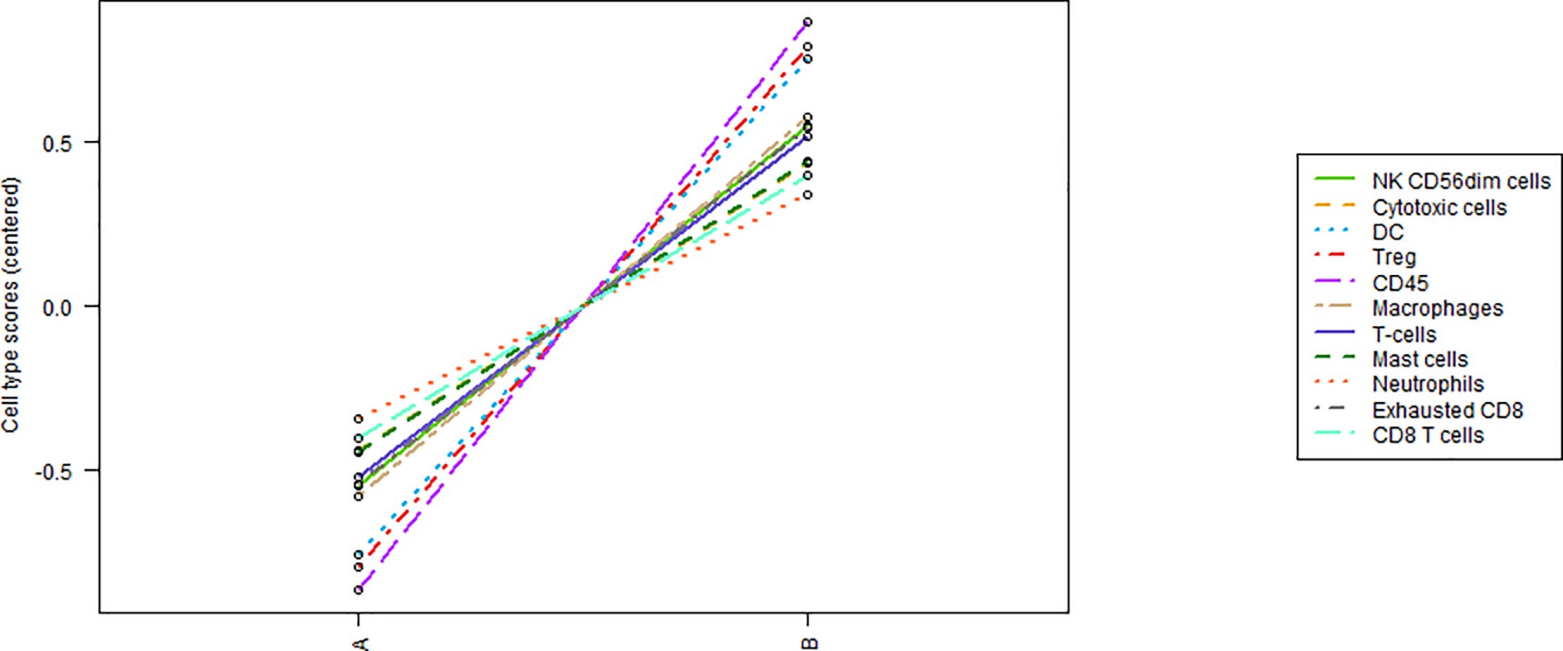
Tumour immune cell profiling by immunohistochemical analysis. Trend plot summarizing the change in abundance of cell types from “worse cases” (A) to “good cases” (B).

**Fig 4:**
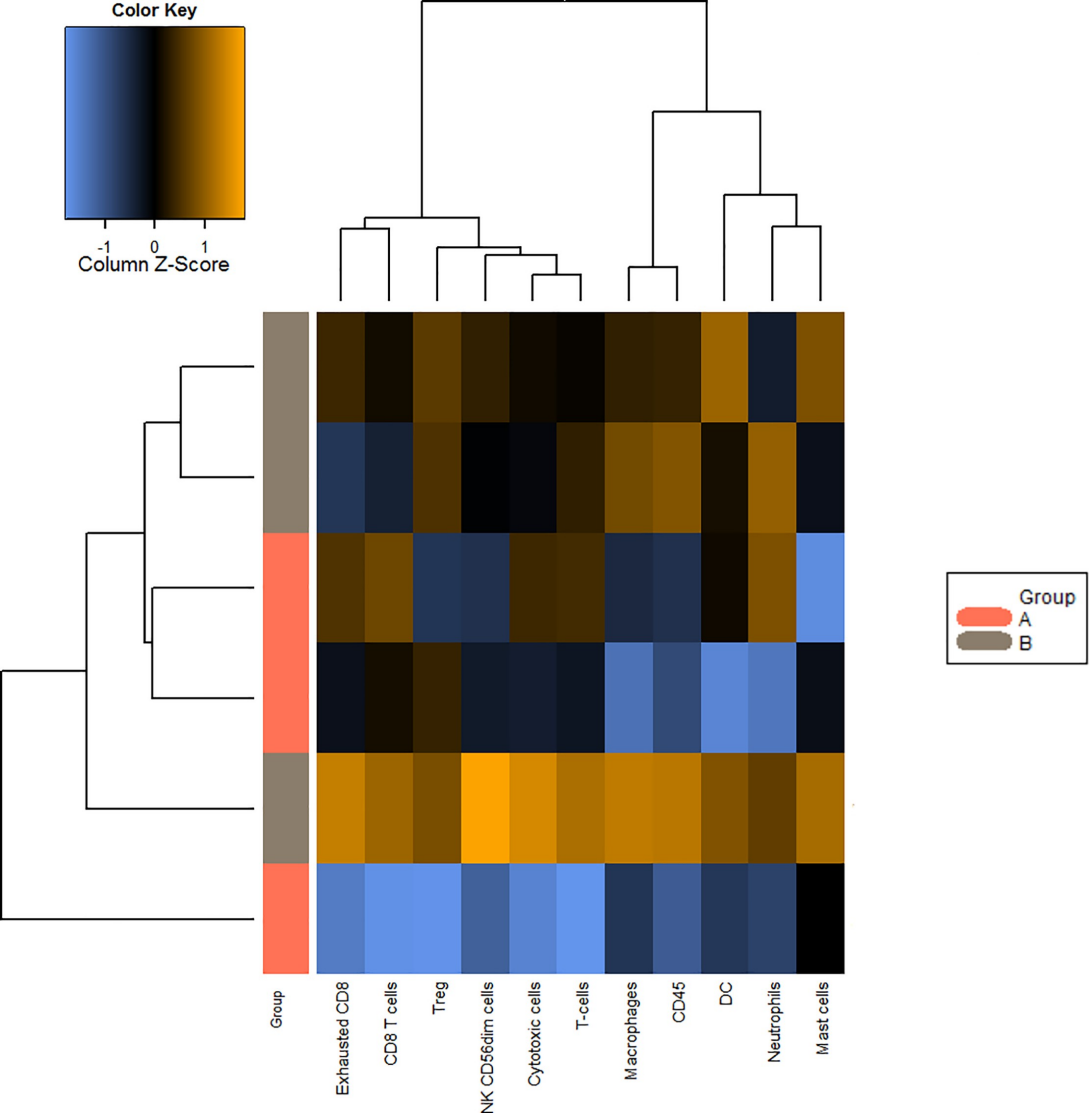
Heat map showing the levels of the different cell types in “worse cases” (pink) and “good cases” (grey). Yellow-orange indicates high abundance, blue indicates low abundance.

**Fig 5:**
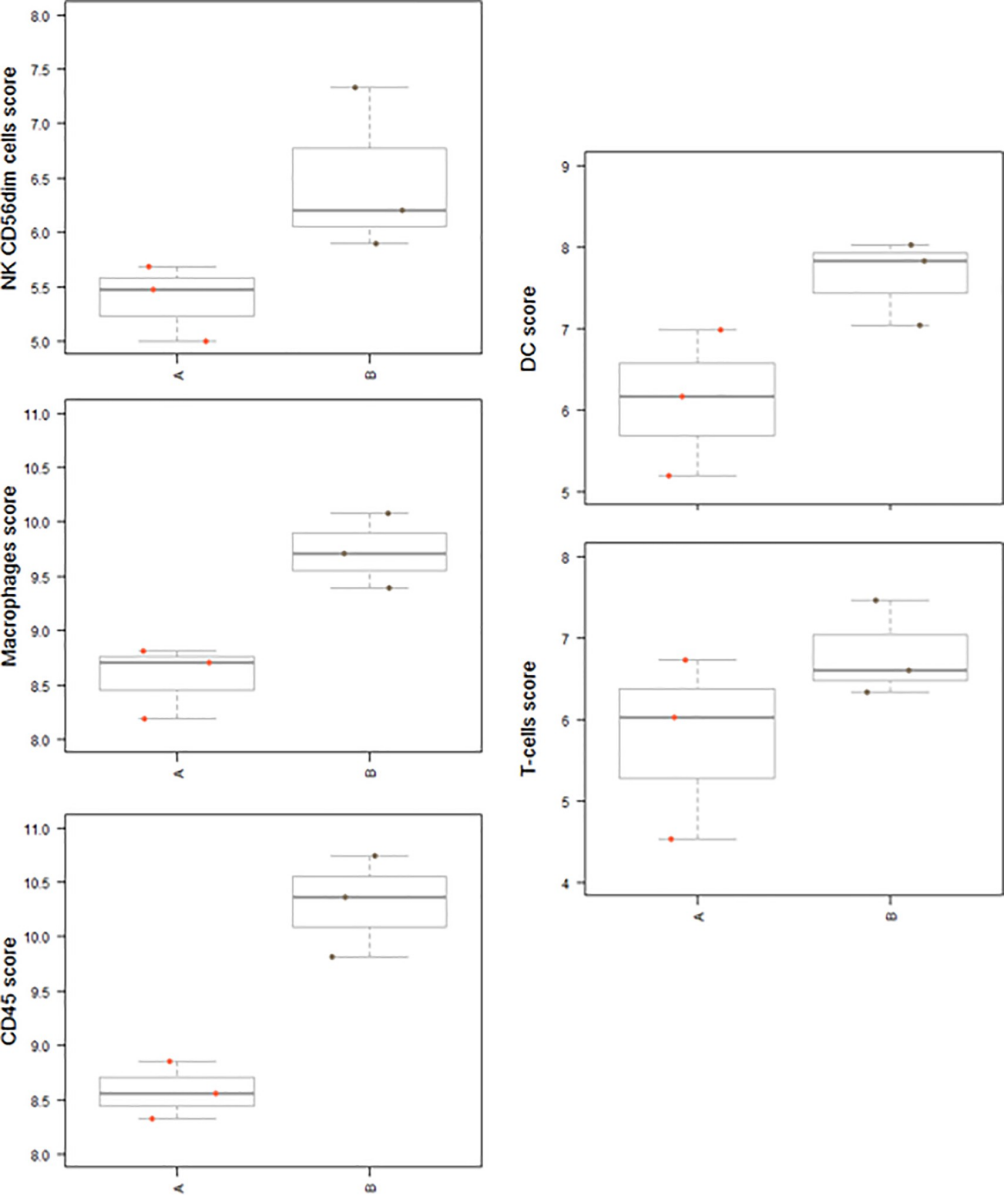
Box plots. Box plots of measurements of CD45-expressing cells, dendritic cells, macrophages, NK, and T cells, in “good cases” (B) and “worse cases” (A).

Gene expression analysis indicated that the pancreatic adenocarcinoma group with a good prognosis showed higher levels of the following cell types compared to the group with a worse prognosis (Figs 3–5): CD45-expressing cells, Tregs, DCs, macrophages, NK CD56dim cells, T cells, exhausted CD8^+^ cells, cytotoxic cells, mast cells, CD8^+^ T cells and neutrophils (Figs 3 and 4). Box plot representations indicate that the following subtypes of cells exhibit particularly different levels: CD45-expressing cells, dendritic cells, macrophages, natural killer cells, the family of T cells (Fig 5), and exhausted CD8^+^ and Treg cells (S1–S3 Figs).

**Table 2:** Top 20 genes differentially expressed between “good cases” and “worse cases”.

Genes	Differential expression between “good cases” and “worse cases” (log2 fold change)	Std error	P-Value
TLR7-mRNA	2.76	0.58	0.00896
TNF-mRNA	2.39	0.389	0.00356
C1QA-mRNA	2.19	0.43	0.00697
FOXP3-mRNA	2.07	0.372	0.00513
CD37-mRNA	2	0.297	0.00254
BTK-mRNA	1.91	0.309	0.0035
CD4-mRNA	1.86	0.235	0.00138
HCK-mRNA	1.86	0.304	0.00364
PTPRC-mRNA	1.83	0.259	0.00211
CCND3-mRNA	1.67	0.337	0.00777
STAT1-mRNA	1.59	0.238	0.00262
IKBKE-mRNA	1.51	0.282	0.00585
IRF8-mRNA	1.43	0.246	0.00439
TNFRSF1A-mRNA	1.39	0.298	0.00954
TLR2-mRNA	1.34	0.147	0.000799
BAX-mRNA	1.31	0.246	0.00598
IRF5-mRNA	1.27	0.193	0.00272
PNMA1-mRNA	0.986	0.201	0.00799
ANP32B-mRNA	0.92	0.163	0.00484
TFE3-mRNA	-0.37	0.0783	0.00919

### Tumour immune cell profiling in pancreatic adenocarcinomas with good versus worse prognosis

**Fig 6:**
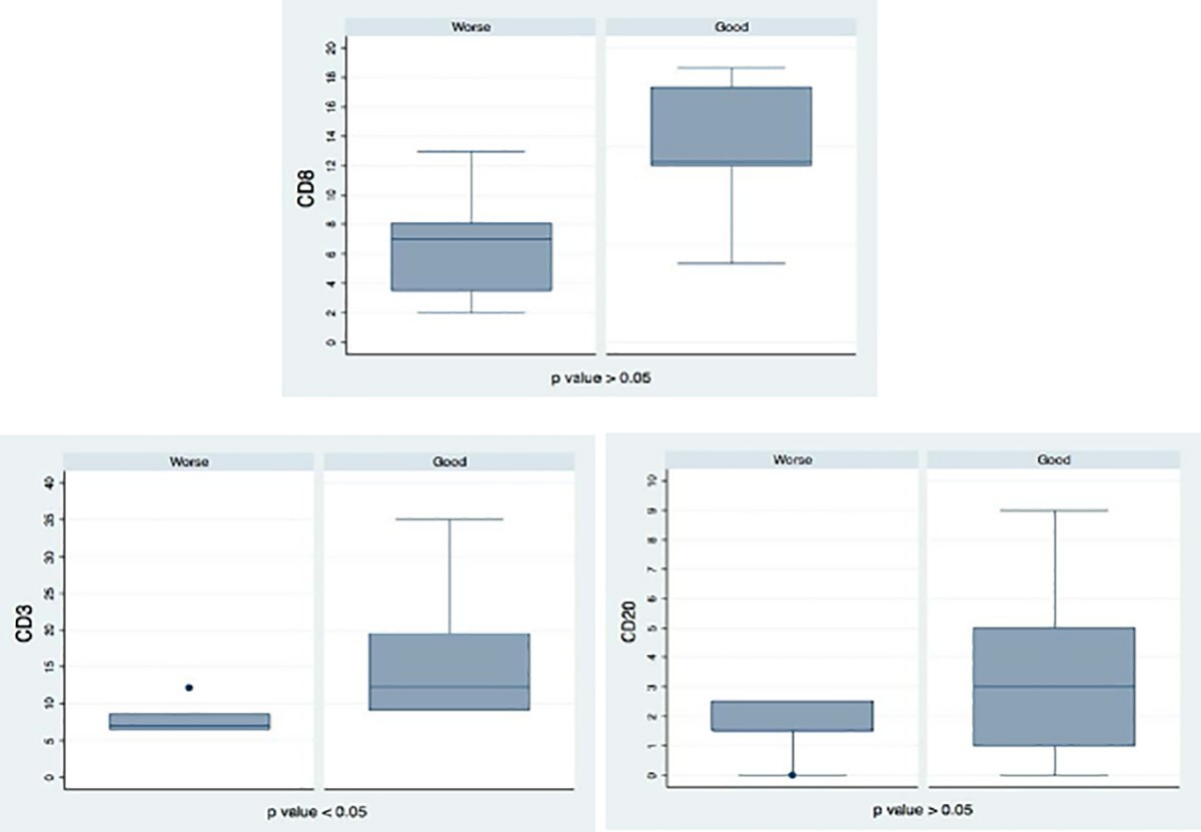
Levels of TIL markers in “worse cases” (Worse) and “good cases” (Good) of pancreatic adenocarcinoma. The box plots represent the expression levels of CD3, CD8 and CD20 TIL subpopulations comparing the “worse case” and “good case” groups. The lower table summarizes the statistical difference of TIL levels between the “worse case” and “good case” groups (non-parametric Mann Whitney test or “U-test”).

**Fig 7:**
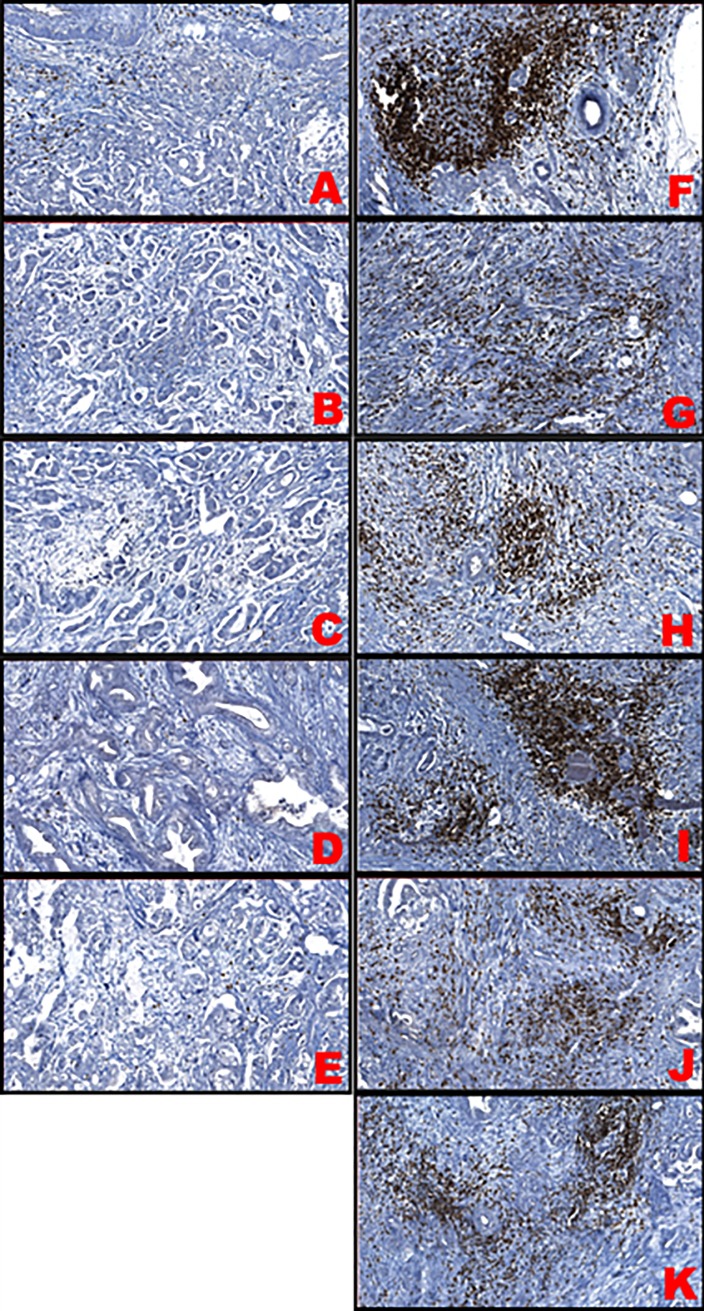
TILs stained for CD3 expression across the two groups of patients. Samples A-E were collected from “worse cases”, samples F-K from “good cases”.

In agreement with existing data [5–9], tumour immune cell marker levels were higher in good prognosis cases compared to worse prognosis cases (Table 3). The CD3 level was statistically higher in the good prognosis group compared to the worse prognosis group (p = 0.0267, Table 4), (Fig 6). Despite the fact that the number of CD8^+^ and CD20^+^ cells has been found to be higher in patients with good prognosis in our study, no statistically significant difference was found between the two subgroups of immune cells (p = 0.119 and p = 0.925, respectively) (Table 4), (Fig 6). TIL marker levels were not assessed in one case due to calcification in pancreatic ductal tissue. Fig 7 shows the detection of TILs in the two sets of six PDAC patients. Staining for TILs was visually negative in the adenocarcinomas with a worse prognosis while it was positive for the adenocarcinomas with a good prognosis.

**Table 3:** Levels of CD3, CD8 and CD20 TIL subpopulations. Data are reported as a percentage value of stroma TILs and are related to the sub-group assessed (CD3, CD8, CD20). For every antigen, two columns of values are reported, each corresponding to a different pathologist’s evaluation. The final score has been given in a semi-quantitative way by evaluating cell density in 10 digital images (20x enlargement) and calculating the average of all scores. The scores have been given following the guidelines of the “International TILs Working Group 2014”.

	Sample number	CD3		CD8		CD20	
**Worse cases**	1	6	7	5	9	1	2
	2	Not performed					
	3	13	11	10	6	2	3
	4	9	8	13	13	1	2
	5	9	4	3	4	2	3
	6	9	5	1	3	0	0
**Good cases**	1	10	8	10	9	2	3
	2	10	8	5	3	0	1
	3	23	16	6	12	0	0
	4	10	8	10	8	0	1
	5	27	16	15	13	5	4
	6	16	15	15	11	2	3

**Table 4:** Statistical difference of TIL levels between the two groups. The table summarizes the statistical difference of TIL levels between the “worse case” and “good case” groups (non-parametric Mann Whitney test or “U-test”).

	Median “worse cases”		Median “good cases”		p-value
	N. pts		N. pts		
CD3	5	7 (6.5–12)	6	12.25 (9–35)	0.0267
CD8	5	7 (2–13)	6	9.25 (4–14)	0.119
CD20	5	1.5 (0–2.5)	6	1.5 (0–4.5)	0.925

## Discussion

Immune cells within the cancer infiltrate may have a role in fighting cancer growth via antigen restricted tumouricidal responses or they may promote tumour progression by suppressing the immune system [15]. There are three major barriers impeding immune therapy in PDAC: 1. The mutational load in PDAC is much lower than that of lung cancers and melanoma; 2. PDAC has a strong immunosuppressive microenvironment which is composed of a dense desmoplastic reaction having remarkable infiltration of tumourigenic MDSCs and macrophages [16]; 3. The PDAC microenvironment has a very low number of infiltrating T cells, insufficient to provide a significant T cell response.

In the current study, PDAC samples from patients with a good prognosis had higher levels of TILs compared to a group of patients with a worse prognosis, as assessed via immune marker levels. Even though the patient numbers are small and the selection of good prognosis or worse prognosis somewhat arbitrary (based on clinical data and Kaplan-Meier curves) (Fig 1), the correlation is consistent with previous reports suggesting that TIL levels provide a robust predictor of outcome in pancreatic cancer [8,17]. Consistent with data reported by *Stromness et al*, we point out that in some samples of the “Good” prognosis group, CD3+ cells tend to organise in tertiary lymphoid structures (TLS) within tumour stroma [18]. Although there is limited knowledge of TLS, these formations are commonly found in solid tumour with a better prognosis, suggesting their possible role in T cell regulation of *in-situ* immune response[18]. Furthermore, our study revealed a significant (p-value <0.001) differential expression of 20 immune system genes between PDAC patients with good and worse prognoses. Among these genes, the expression of five (*TLR7*, *TNF*, *C1QA*, *FOXP3*, *CD37*) was at least twice log 2 higher in the good prognosis group relative to the worse prognosis group. Expression levels of these five genes could constitute a molecular signature of likely outcome and could therefore be useful for clinical applications.

*FOXP3* is a well known marker of Tregs, with a pivotal role in the development and differentiation of these cells to promote tumour immune escape [19,20]. Conversely, *FOXP3* has been reported to be an important tumour suppressor gene in breast cancer [21–25], gastric adenocarcinoma [26,27], prostate cancer [28], and non-small cell lung cancer [29]. These findings indicate that the roles of *FOXP3* in tumours are diverse and situation-dependent.

*C1QA* encodes the A-chain polypeptide of complement subcomponent C1q and plays an important role in counteracting tumour cells [30,31]. Teschendorff and Caldas et al showed that overexpression of *C1QA* in ER-negative basal-like breast cancer patients is associated with better prognosis [32]. It was shown more recently that lower *C1QA* expression could be linked with worse outcomes in patients with ER-negative breast cancer [33]. Nonetheless, Bulla et al recently showed that C1q can exert functions unrelated to complement activation, contributing to extracellular changes within the tumour microenvironment and supporting tumour growth and invasion [34]. This last finding is supported by Winslow et al [35].

TNF has long been considered a key regulator of the inflammatory and immune response to cancer, promoting either death or survival under different circumstances [36]. Although several anti-TNF therapies have been developed with different binding and pharmacokinetic profiles [37], TNF is used in current therapies to fight cancer, notwithstanding its toxicity [38]. TNF has proved to have an effect on metastatic melanoma treatment and unresectable soft tissue therapies [39,40]. There is evidence of TNF’s role in promoting regression of unresectable hepatic metastasis from colorectal cancer [41] and in causing tumour necrosis via its pro-coagulant effect [42].

TLR7 is of special interest in cancer therapy on account of its strong stimulation of IL-12 and type-I interferons, which are important cytokines and effectors of T and NK cell functions [43,44]. TLR7 ligands can not only activate directly NK cells and cytotoxic T-cells [45,46], but also hamper the suppressive function of myeloid-derived suppressor cells [47,48] and interfere with the migration of Tregs into the tumour [49]. TLR agonists are clinically approved or under clinical evaluation for cancer immunotherapy [50–52].

CD37 belongs to the tetraspanin superfamily of transmembrane proteins that regulate protein adhesion, trafficking, and migration and that are emerging controllers of both humoral and immune control, especially stimulating dendritic cell migration and B cell survival [53–55]. The contribution of CD37 to antitumour immunity has been known since the finding that CD37^-/-^ mice have impaired antitumour responses [56]; however, the role of CD37 in the tumour microenvironment is not clear and further investigations are needed. Tetraspanins in the tumour microenvironment may have therapeutic potential via stimulation or inhibition of immune cell functions, depending on the immune cell type [57].

There are numerous biomarkers that have proved to be clinically useful for other cancers such as lung cancer [58], colorectal cancer [59], breast cancer [60] and melanoma [61], but clinical application of biomarkers for PDAC has been somewhat limited. Indeed, the only FDA-approved PDAC marker, the serum protein CA 19–9, was approved in the 1980s [62]. At least 10% of patients do not express CA 19–9, however, and its level is easily affected by metabolic abnormalities. Several studies have identified biomarkers that could be used as predictors of clinical outcome for PDAC [63–66], but none of these involves the immune-related gene signature revealed here.

The key findings from this study, that longer-surviving PDAC patients had higher levels of intratumoural TILs and overexpressed five immune markers (*TLR7*, *TNF*, *C1QA*, *FOXP3*, *CD37*), could have two main uses. Firstly, TIL levels and marker gene panel expression could be used for clinical outcome prediction, stratification and treatment design for PDAC patients. A previous study showed that a signature comprising another 15 genes was an independent prognostic factor in two cohorts of PDAC patients. In contrast to our results, higher expression of these 15 genes was associated with poor OS [63]. Similarly, Sergeant et al identified high co-expression of *TGF-β1* and a panel of cell motility genes as independent predictors of worse clinical outcome [64], while Van den Broek et al discovered that high expression of *ABCB1* and *CXCR4* correlated with worse clinical outcome [65]. Furthermore, decreased levels of *DPEP1* and increased expression of *TPX2* were independently associated with poor survival [66]. Presumably, a wide panel of validated gene signatures would be most useful for outcome prediction, stratification and therapeutic decision making.

Secondly, our findings could be useful in developing new PDAC treatments, for example in combination with current immunotherapeutic strategies. Expression of the target genes identified here could be induced together with therapies modulating the tumour microenvironment to relieve immunosuppression, and/or approaches to break down the desmoplastic barrier surrounding PDAC to facilitate target access for infiltrating T cells or therapeutic molecules [15]. Such strategies could be effected in combination with recently reported gene therapy and oncologic vaccination approaches [67–69].

In summary, our data indicate that a gene signature comprising at least *TLR7*, *TNF*, *C1QA*, *FOXP3*, and *CD37* could be useful to improve the prediction of OS in PDAC patients. Together with an assessment of TIL levels, such an immune system gene panel constitutes a potential prognostic tool to permit a risk-based stratification of pancreatic tumour patients into personalized treatment protocols towards improving the current abysmal clinical outcome of these patients.

### Future perspective

The findings in the paper might be useful in stratifying patients and indicating the best treatment available for pancreatic cancer patients. In addition, our study could pave the way for finding novel targets for the development of new drugs for this disease.

## Supporting information

S1 Fig Differential gene expression between the good and worse prognosis PDAC patient groups. Volcano plot displaying each gene's -log10(p-value) against log2 fold change: a) *TLR7*, b) *TNF*, c) *C1QA*, d) *FOXP3* and e) *CD37*. Highly statistically significant genes fall at the top of the plot, and highly differentially expressed genes fall to either side. Genes within the selected gene set are highlighted in orange. Horizontal lines indicate various False Discovery Rate (FDR) thresholds.
(TIFF)

S2 Fig Exhausted CD8^+^ and Treg cell profiling in pancreatic adenocarcinomas with good versus worse prognosis. Box plots show levels of exhausted CD8^+^ cells (a) and Tregs (b) in patients with worse prognosis (group A) and patients with worse prognosis (group B). Even though scores seem overlapping, the average score for both sets of cells is higher in group B than in group A, probably due to the fact that longer-surviving PDAC patients had higher levels of intratumoral TILs.
(TIFF)

S3 Fig Relative cell type abundance measurements between group A and B. The diagram shows the abundance of exhausted CD8^+^ cells and Tregs compared to levels of CD8^+^ cells. In agreement with the previous figure, levels of exhausted CD8^+^ cells (green line) and Tregs (dashed orange line) are reported to be lower in the group with a worse prognosis (group A) than in the group with a better prognosis (group B) when compared with the total level of CD8^+^ cells.
(TIFF)

S1 Table Dataset used for Kaplan-Meyer curves. (PDF)
