# Supplementary figures and images for: Tumour infiltrating lymphocytes and immune-related genes as predictors of outcome in pancreatic adenocarcinoma
Source: PLoS One. 2019 Aug 5;14(8):e0219566. doi: 10.1371/journal.pone.0219566 (PMC6681957; doi:10.1371/journal.pone.0219566)

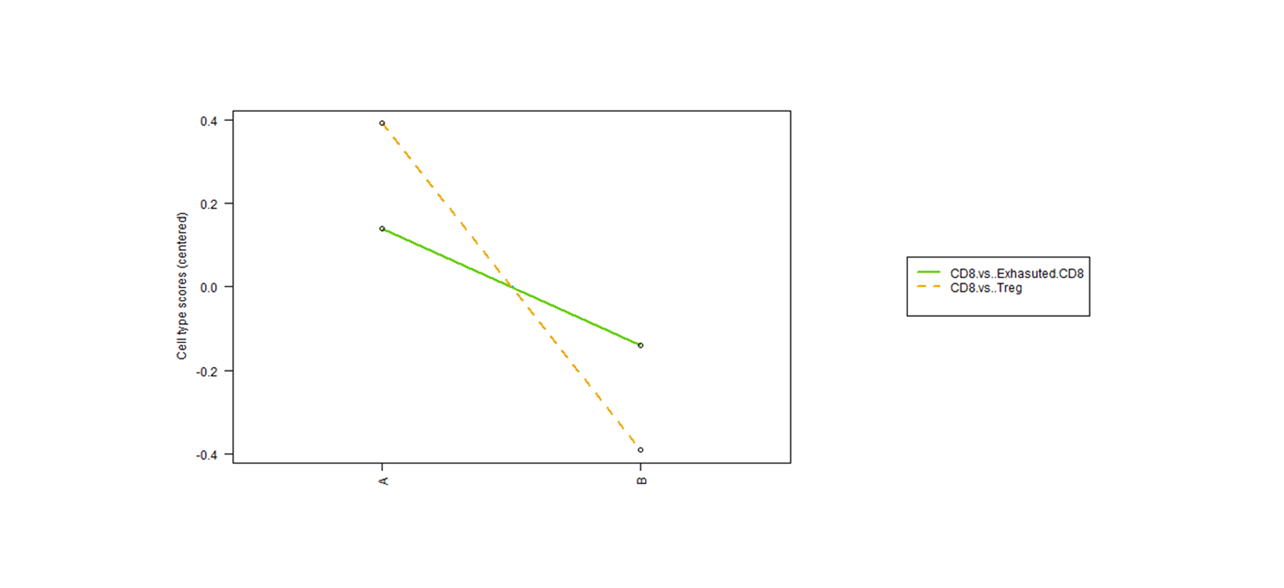

Supplement: S1 Fig — Volcano plot displaying each gene's -log10(p-value) against log2 fold change: a) TLR7, b) TNF, c) C1QA, d) FOXP3 and e) CD37. Highly statistically significant genes fall at the top of the plot, and highly differentially expressed genes fall to either side. Genes within the selected gene set are highlighted in orange. Horizontal lines indicate various False Discovery Rate (FDR) thresholds. (TIFF) [file pone.0219566.s001.tiff]

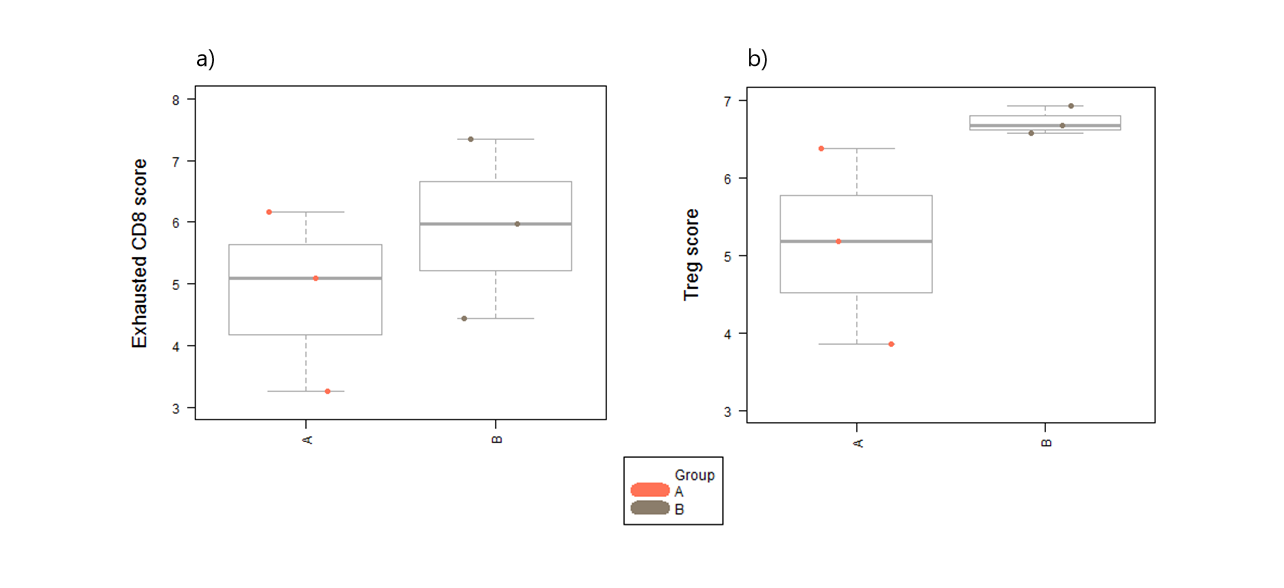

Supplement: S2 Fig — Box plots show levels of exhausted CD8+ cells (a) and Tregs (b) in patients with worse prognosis (group A) and patients with worse prognosis (group B). Even though scores seem overlapping, the average score for both sets of cells is higher in group B than in group A, probably due to the fact that longer-surviving PDAC patients had higher levels of intratumoral TILs. (TIFF) [file pone.0219566.s002.tiff]

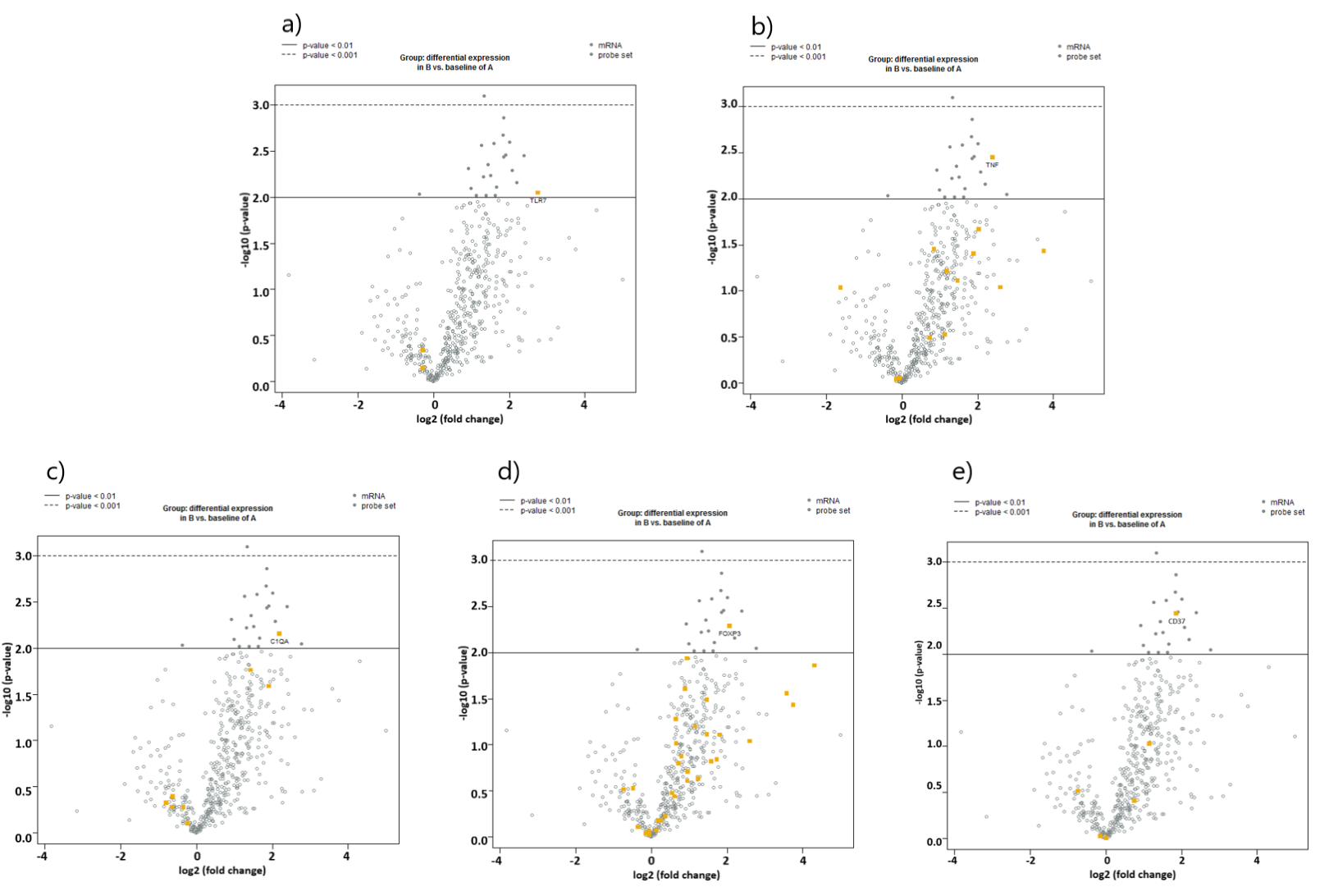

Supplement: S3 Fig — The diagram shows the abundance of exhausted CD8+ cells and Tregs compared to levels of CD8+ cells. In agreement with the previous figure, levels of exhausted CD8+ cells (green line) and Tregs (dashed orange line) are reported to be lower in the group with a worse prognosis (group A) than in the group with a better prognosis (group B) when compared with the total level of CD8+ cells. (TIFF) [file pone.0219566.s003.tiff]
