# Supplementary material for: Tumour infiltrating lymphocytes and immune-related genes as predictors of outcome in pancreatic adenocarcinoma
Source: PLoS One. 2019 Aug 5;14(8):e0219566. doi: 10.1371/journal.pone.0219566 (PMC6681957; doi:10.1371/journal.pone.0219566)
Supplement: S1 Table — (PDF) [file pone.0219566.s004.pdf]

ID	date diagnosis	date of metastasis	DFS (months)	exitus	date of death	OS (months)
Worse cases	18/09/2013	21/11/2013	2	yes	07/01/2014	4
	08/11/2013	20/01/2014	2	yes	10/08/2014	9
	16/01/2014	20/02/2014	1	no		
	16/02/2015	08/04/2015	2	yes	25/07/2015	5
	22/06/2015	27/08/2015	2	yes	25/09/2015	3
	26/10/2015	06/11/2015	1	no		
Good cases	05/03/2008	01/12/2011	45	yes	07/03/2012	48
	19/05/2009	01/10/2010	17	yes	05/09/2012	40
	22/09/2009	01/04/2014	55	yes	17/03/2015	66
	20/10/2011	28/05/2012	7	yes	29/09/2014	35
	21/02/2013	29/10/2013	8	yes	07/03/2015	25
	27/01/2009	29/09/2013	56	yes	03/07/2014	66
